# Bone-Healing Capacity of PCL/PLGA/Duck Beak Scaffold in Critical Bone Defects in a Rabbit Model

**DOI:** 10.1155/2016/2136215

**Published:** 2016-03-03

**Authors:** Jae Yeon Lee, Soo Jin Son, Jun Sik Son, Seong Soo Kang, Seok Hwa Choi

**Affiliations:** ^1^College of Veterinary Medicine, Chungbuk National University, Cheongju 362-763, Republic of Korea; ^2^Korean Textile Development Institute, Daegu 703-712, Republic of Korea; ^3^College of Veterinary Medicine, Chonnam National University, Gwangju 500-757, Republic of Korea

## Abstract

Bone defects are repaired using either natural or synthetic bone grafts. Poly(*ϵ*-caprolactone) (PCL), *β*-tricalcium phosphate (TCP), and poly(lactic-co-glycolic acid) (PLGA) are widely used as synthetic materials for tissue engineering. This study aimed to investigate the bone-healing capacity of PCL/PLGA/duck beak scaffold in critical bone defects and the oxidative stress status of the graft site in a rabbit model. The* in vivo* performance of 48 healthy New Zealand White rabbits, weighing between 2.5 and 3.5 kg, was evaluated. The rabbits were assigned to the following groups: group 1 (control), group 2 (PCL/PLGA hybrid scaffolds), group 3 (PCL/PLGA/TCP hybrid scaffolds), and group 4 (PCL/PLGA/DB hybrid scaffolds). A 5 mm critical defect was induced in the diaphysis of the left radius. X-ray, micro-CT, and histological analyses were conducted at (time 0) 4, 8, and 12 weeks after implantation. Furthermore, bone formation markers (bone-specific alkaline phosphatase, carboxyterminal propeptide of type I procollagen, and osteocalcin) were measured and oxidative stress status was determined. X-ray, micro-CT, biochemistry, and histological analyses revealed that the PCL/PLGA/duck beak scaffold promotes new bone formation in rabbit radius by inducing repair, suggesting that it could be a good option for the treatment of fracture.

## 1. Introduction

Three-dimensional (3D) polymeric scaffolds are used as bone grafts for repairing a variety of large bone defects. Multihead deposition system (MHDS), a type of solid freeform fabrication (SFF) technology, fabricates 3D scaffolds using polycaprolactone (PCL) and poly(lactic-co-glycolic) acid (PLGA), which are widely used in bone tissue engineering [1, 2].

Alternatively, bone defects can also be treated using xenogenous bone, which is made of animal bone and is cost-effective and readily available in large supply. Studies have reported the use of bones of animals and fish, such as cow, tuna, and cod, for developing bone scaffolds [3]. Duck beaks are one of the lowest quality parts of the animal (head, beaks, feet, etc.) and are not used as food, natural organic fertilizer, and animal feed. Therefore, duck beak can be used as a biomaterial for fabricating bone scaffolds. In this study, duck beaks were sintered and ground into micropowder and were used as ceramic biomaterial in a novel scaffold that could be used for cell growth and tissue regeneration.

Oxidative stress is a biochemical disequilibrium that is caused by excessive production of free radicals and reactive oxygen species (ROS), which induce oxidative damage to biomolecules; this oxidative stress damage cannot be counteracted by antioxidative systems [4]. ROS, such as hydroxyl radical and superoxide radical, are reactive chemical species generated during normal metabolic processes, but, in excess, they can damage lipids and proteins. Oxidative stress results from a chain of oxidative events that leads to increased production of ROS, which causes tissue injury. Following a fracture, oxidative stress injury may be caused by an ischemia-reperfusion mechanism [5, 6]. Bone markers, such as osteocalcin and alkaline phosphatase, play a significant role in the healing of bone fractures, whereas oxidative stress delays healing [7–9].

This study aimed to investigate the bone-healing capacity of PCL/PLGA/duck beak scaffold in critical bone defects and the oxidative stress status of the graft site in a rabbit model.

## 2. Materials and Methods

### 2.1. Animals

Forty-eight healthy New Zealand White rabbits, weighing between 2.5 and 3.5 kg, were included in this study. The protocol for the animal experiment was approved by the Laboratory Animal Research Center of Chungbuk National University (CBNUA-677-14-01). All rabbits were kept in individual cages throughout the experimental period. Water and food were supplied ad libitum during the experimental period. Rabbits were assigned to the following groups: group 1 (control, only with critical bone defect), group 2 (PCL/PLGA hybrid scaffolds), group 3 (PCL/PLGA/TCP hybrid scaffolds), and group 4 (PCL/PLGA/duck beak hybrid scaffolds). Four rabbits from each group (total 12 rabbits per group) were sacrificed at 4, 8, and 12 weeks after implantation. Serum samples were collected before surgery (time 0) and 4, 8, and 12 weeks after surgery for evaluation of biochemical markers.

### 2.2. Preparation of Blended PCL/PLGA and PCL/PLGA/TCP

PCL (19561-500G, MW 43,000–50,000; Polysciences Inc., Warrington, PA, USA), PLGA (430471-5G, MW 50,000–75,000; Sigma-Aldrich, St. Louis, MO, USA), and TCP (average diameter 100 nm; Berkeley Advanced Biomaterials Inc., Berkeley, CA, USA) were blended together using melting process [10]. Briefly, granular PCL (0.4 g) and PLGA (0.4 g) were placed on a glass container and melted at 130°C for 10 min. The molten PCL and PLGA were manually mixed to create blended PCL/PLGA. The blended PCL/PLGA/TCP was prepared by adding powdered b-TCP (0.2 g) to the molten state of the blended PCL/PLGA. The b-TCP was blended with PCL/PLGA for 5 min.

### 2.3. Fabrication of PCL/PLGA and PCL/PLGA/TCP Scaffolds Using MHDS

The blended PCL/PLGA polymer and PCL/PLGA/TCP polymer were fed to a 10 cc syringe and melted by heating for 10 min to their melting temperature. Then, the biodegradable polymer was extruded from the nozzle by controlling the pneumatic pressure and temperature of the syringe. The deposition characteristics are very sensitive to the major process parameters, such as pressure, temperature, and moving velocity. In this study, the effect of pressure, temperature, and moving velocity was investigated through repetitive experiments according to the various ranges of process parameters. Among the conditions of process parameters, PCL/PLGA and PCL/PLGA/TCP scaffold having the line width and height of 200 *μ*m were fabricated at pressure of 650 kPa, temperature of 120°C, and* X*-*Y* moving velocity of 35 mm/min. Stacked scaffolds with a staggered shape were constructed using a layer-by-layer process to achieve an overall scaffold size of 3 × 3 × 20 mm.

### 2.4. Preparation of Duck Beak Powder and Composite Bone Scaffolds

Bone powder was obtained from livestock duck (cherry valley, 6–8 weeks old) beaks which were raised in local farms. The duck beak was soaked in oxygenated water for 48 h to remove the surface impurities. It was then cut into rectangular samples of approximate size 10 mm × 10 mm × 10 mm. The samples were sintered in an electric furnace (ST-01045, Daihan Scientific, Korea) at 1100°C for 2 h to eliminate the organic compounds of the samples. The sintered duck beaks were pulverized by a miller (A10, IKA-WERKE, Japan). Particle size of sintered bone powder was classified using sieves of 150–200 *μ*m (Sieve/Shaker, Daihan Scientific, Korea). The sintered duck beaks were then sterilized in an autoclave. Composite polymer solution was made by mixing PCL (0.4 g), PLGA (0.4 g), and powdered duck beak (0.2 g). The molten PCL and PLGA were manually mixed to create blended PCL/PLGA. The blended PCL/PLGA/duck beak was prepared by adding powdered duck beak (0.2 g) to the molten state of the blended PCL/PLGA. The powdered duck beak was blended with PCL/PLGA for 5 min. The heat-treated beak bone particles have many pores of macro- and micrometersize, having a porosity of 77.3 ± 2.6% and a pore size of 2.787 ± 0.10 *μ*m. The Ca/P atomic ratio of beak bone particle was 1.65 and did not contain any distinguishable crystalline impurity, and this bone particle was crystallized HA with high crystallinity by the heating process. These data were presented by our previous study [11].

### 2.5. Scanning Electron Microscope (SEM) and Energy Dispersive Spectrometer (EDS) Analysis of Scaffolds

The morphology of the cylinder-type scaffolds was observed using a field emission (FE) SEM (SEM; JSM-5300, JEOL, Japan) at 10 kV. The surface morphology of the PCL/PLGA/duck beak scaffold was compared with that of the PCL/PLGA and PCL/PLGA/TCP scaffold. The calcium and phosphorus ratio was measured with an energy dispersive spectrometer (EDS, Inca x-sight, Oxford Instruments, UK).

### 2.6. Surgery

Surgery was performed under general anaesthesia. Rabbits were anaesthetised with a cocktail of 35 mg/kg ketamine and 5 mg/kg xylazine administered intramuscularly. Prior to the surgery, the skin was shaved and then cleaned with a mixture of iodine and 70% ethanol. The operation site over the radius was exposed after skin incision. An approximately 4 cm longitudinal medial incision was made and the tissues overlying the mid-diaphyseal radius were dissected. The radius, together with periosteum, was completely sawed off at 20 mm distal to the head of radius with a wire saw under constant irrigation, and then the radius was sawed off at 20 mm distal to the first osteotomy line. The scaffolds were inserted in the defect without external fixation. All surgeries were conducted by the same investigator. After surgery, animals were radiographed immediately. Enrofloxacin (10 mg/kg, sc, bid) and ketoprofen (3 mg/kg, im, sid) were given for pain relief and infection prophylaxis.

### 2.7. Radiographic and Microcomputed Tomographic (CT) Evaluation

Four rabbits from each group were euthanized at 4, 8, or 12 weeks after surgical procedures, after X-ray images were taken with an X-ray machine (Rotanode; Toshiba, Japan) from a distance of 100 cm (60 kVp and 300 mA) with an exposure time of 0.03 sec. Digital images were used to evaluate the degree of bone healing based on the criteria described by Cook et al. [12]. The specific scores were as follows: no visible new bone formation, 0; minimal new disorganized bone, 1; disorganized new bone bridging grafted to host at both ends, 2; organized new bone of cortical density bridging at both ends, 3; loss of graft-host distinction, 4; and significant new bone and graft remodelling, 5. After X-ray images were taken, the radiuses were collected and fixed in 10% neutral buffered formalin. Three bone graft substitutes and samples taken at 4, 8, and 12 weeks after implantation were imaged using a micro-CT (SkyScan Desktop Micro-CT 1172; SkyScan, Belgium). The scanned data were reconstructed using a software (NRecon; SkyScan). Bone volume fraction (%) of the three type scaffolds was calculated according to the program set by the software. Grey thresholds were set from 65 to 255 using image analysis software (CT-analyser; SkyScan).

### 2.8. Biochemical Parameters

#### 2.8.1. Blood Sample Collection

Blood samples were collected in the morning 1 to 2 h before surgery (time 0) and at 4, 8, 12, and 16 weeks after surgery. The samples were collected in 12 mL volume syringes via a 20-gauge catheter placed in the auricular artery and then immediately transferred to a serum collection tube, placed on ice, and then refrigerated (4°C). Following clot formation, samples were centrifuged at 4°C, and serum aliquots were stored at –80°C. All serum samples were analysed at the completion of the study.

#### 2.8.2. Measurement of Bone Formation Markers

Bone-specific alkaline phosphatase (BALP, BSALP ELISA Kit, Cusabio Biotech, Co. Ltd., Wuhan, China), carboxyterminal propeptide of type I procollagen (PICP, Rabbit Carboxyterminal Propeptide of Type I Procollagen ELISA Kit, Cusabio Biotech, Co. Ltd., Wuhan, China), and osteocalcin (OC, Rabbit Osteocalcin ELISA Kit, Cusabio Biotech, Co. Ltd., Wuhan, China) activities were measured using an enzyme linked immunosorbent assay (ELISA).

#### 2.8.3. Measurement of Total Oxidant Status (TOS)

Blood samples were centrifuged at 3000 rpm for 10 min to separate plasma. The collected plasma samples were then stored at −80°C until analysis. Plasma TOS was determined using a commercially available kit developed by Erel [13]. TOS is expressed as micromolar hydrogen peroxide equivalents per litre (*μ*mol H_2_O_2_ equiv/L).

### 2.9. Histomorphometry

The specimen including the implant was prepared after the experimental animals were sacrificed. The specimen was fixed for 2 weeks in a neutral buffered formalin solution (Sigma-Aldrich, St. Louis, MO, USA) and then dehydrated by increasing the ethanol concentration from 70 to 100%. The recovered formalin-fixed alcohol-preserved specimens were decalcified in 10% formic acid/formalin solution for 14 days, dehydrated, paraffin-embedded, microsected parallel to the bone axes, and stained with hematoxylin and eosin (H&E) and Masson's trichrome. Histological observation of the grafted bone granule resorption, degree of regenerated bone replacement, and the inflammatory response were recorded using Olympus BX40 microscopy (Olympus, Tokyo, Japan).

### 2.10. Statistical Analysis

Statistical analyses were performed using SPSS statistical software package version 19.0.1.1. Data are presented as the mean ± standard deviation (SD). Normality and homogeneity of the data were confirmed before analysis of variance (ANOVA). Differences among the experimental groups were assessed by one-way ANOVA followed by Duncan's multiple range tests. *P* < 0.05 was considered statistically significant.

## 3. Results

### 3.1. Material Property and Mechanical Strength of Each Scaffold

**Figure 1:**
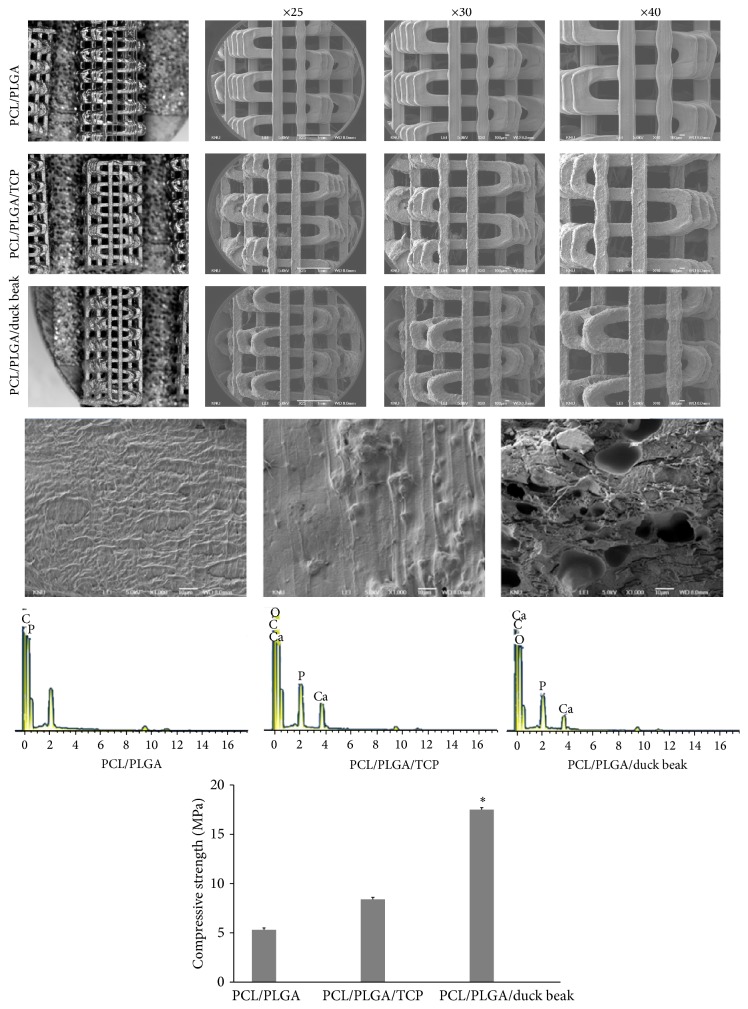
Stereo microscope and scanning electron microscope (SEM) images, energy dispersive X-ray spectroscopy (EDS) spectrum and compressive strength of PCL/PLGA, PCL/PLGA/TCP, and PCL/PLGA/duck beak scaffolds. ^*∗*^Significantly different (*P* < 0.05) from PCL/PLGA and PCL/PLGA/TCP.

Figure 1 shows the SEM image of PCL/PLGA, PCL/PLGA/TCP, and PCL/PLGA/duck beak scaffolds. The PCL/PLGA/duck beak scaffold had an irregular oblong shape with several macro- and micropores compared with PCL/PLGA and PCL/PLGA/TCP scaffolds. The pores were irregularly arranged with elongation along layers. In addition, the surface of PCL/PLGA/TCP scaffold had rough, compact, and dense structures within interconnected mesopores distributed over its entire surface, as shown in Figure 1. Figure 1 shows the EDS spectrum of scaffolds. The atomic ratio Ca/P was 1.67 and 1.65 for TCP and duck beak incorporated scaffold. The EDS study revealed that beak bone particles were composed of Ca and P and that the atomic ratio of Ca/P was 1.65, indicating a similar atomic ratio to that of human bone (Ca/P ratio: 1.50–1.70). These data were presented by our previous study [11]. In addition, the compressive strength of PCL/PLGA/TCP scaffold was significantly higher than that of PCL/PLGA and PCL/PLGA/TCP scaffolds (Figure 1).

### 3.2. Bone-Healing Effects of PCL/PLGA, PCL/PLGA/TCP, and PCL/PLGA/Duck Beak Scaffolds by Radiographic Analysis

Different scaffolds were implanted and X-ray images were taken at 0, 4, 8, and 12 weeks following the induction of 20 mm segmental bone defects of radiuses. Callus formation, but no union, was observed in the untreated rabbits at 12 weeks after the surgery. PCL/PLGA scaffold was similar to the no treatment group at 0 weeks after implantation due to its low radiopacity.

**Figure 2:**
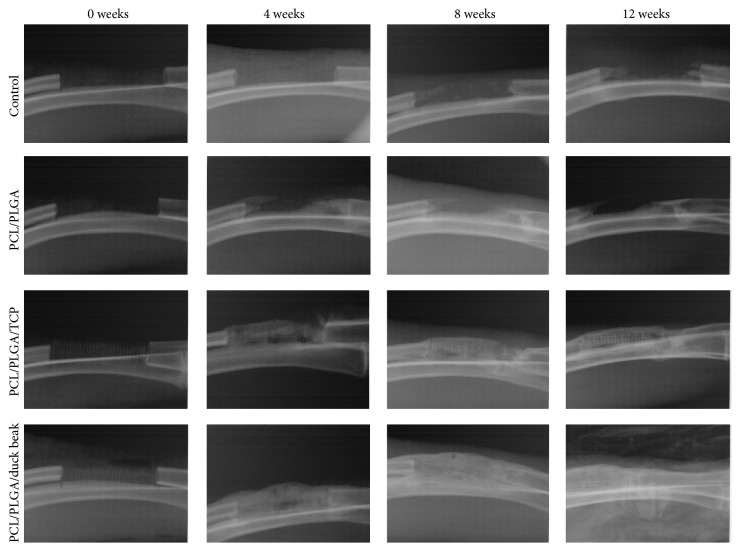
Radiographic images of no treatment, PCL/PLGA hybrid scaffolds, PCL/PLGA/TCP hybrid scaffolds, and PCL/PLGA/duck beak hybrid scaffolds at 0, 4, 8, and 12 weeks after implantation. There was callus formation but no union in the control group at 12 weeks after the surgery. PCL/PLGA hybrid scaffold was similar to the control group at 0 weeks after implantation due to its low radiopacity. There were increased new bone densities, but no difference was observed in PCL/PLGA/TCP hybrid scaffold and PCL/PLGA/duck beak hybrid scaffold at 0, 4, 8, and 12 weeks after implantation.

**Figure 3:**
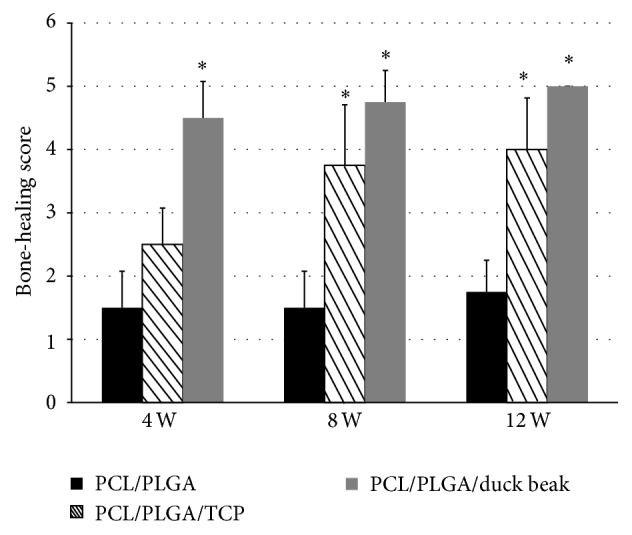
Bone-healing scores measured by radiographic image analysis. Four rabbits from each group were euthanized at 4, 8, or 12 weeks after surgical procedures, respectively, and X-ray images were taken using an X-ray machine. Digital images were used to evaluate the degree of bone healing based on the criteria defined by Cook et al. [5]. Bone-healing scores increased in the PCL/PLGA/TCP and PCL/PLGA/duck beak scaffolds groups at 8 and 12 weeks after implantation. The values shown are the mean ± SD (*n* = 4). ^*∗*^Significantly different (*P* < 0.05) from PCL/PLGA.

The bone formation in duck beak group is significantly better than TCP groups at 4 weeks from imaging results. Increased new bone density was observed in PCL/PLGA/duck beak scaffold groups at 4, 8, and 12 weeks after implantation and in PCL/PLGA/TCP groups at 8 and 12 weeks after implantation (Figure 2). Significant difference was observed in PCL/PLGA/TCP and PCL/PLGA/duck beak scaffold groups at 4 weeks, but no difference was observed at 8 and 12 weeks after implantation. Radiographic analysis showed increased bone-healing scores in the PCL/PLGA/TCP and PCL/PLGA/duck beak scaffold groups at 8 and 12 weeks after implantation, as shown in Figure 3.

### 3.3. Micro-CT Findings

**Figure 4:**
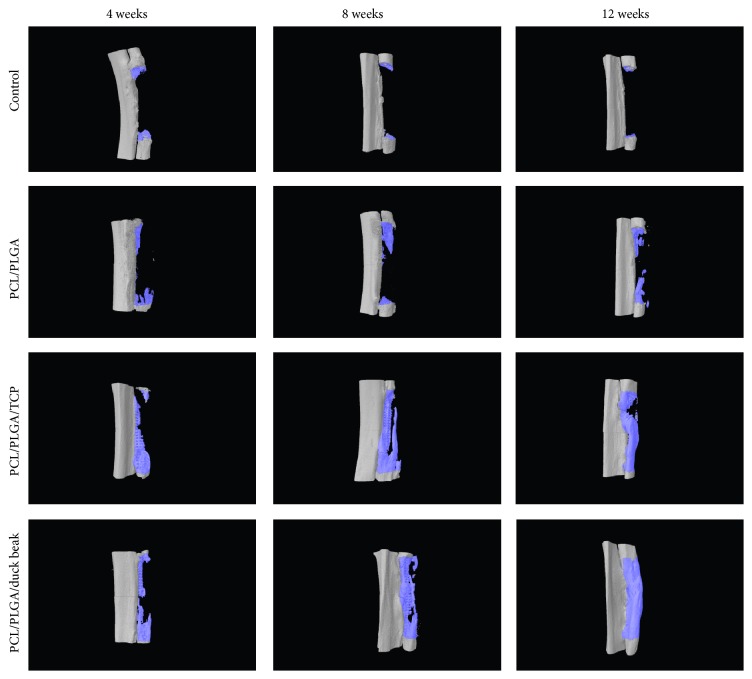
Micro-CT images of no treatment, PCL/PLGA hybrid scaffolds, PCL/PLGA/TCP hybrid scaffolds, and PCL/PLGA/duck beak hybrid scaffolds at 0, 4, 8, and 12 weeks after implantation. There was callus formation but no union in the control group at 12 weeks after the surgery. PCL/PLGA hybrid scaffold was similar to the control group at 0 weeks after implantation due to its low radiopacity. There were increased new bone densities, in PCL/PLGA/TCP hybrid scaffolds and PCL/PLGA/duck beak hybrid scaffolds at 8 and 12 weeks after implantation.

**Figure 5:**
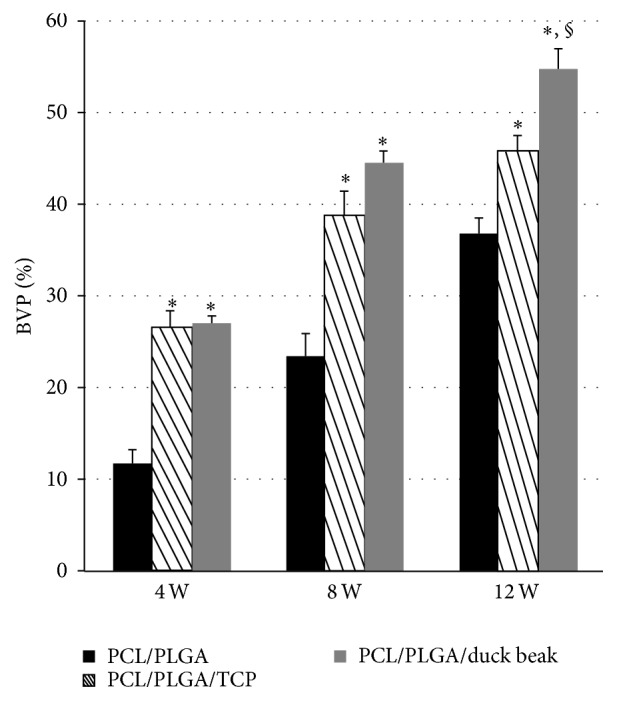
Changes in bone volume fraction (%) after implantation. The samples from the euthanized rabbits were imaged using a micro-CT at 4, 8, and 12 weeks after implantation. The scanned data were reconstructed using software. Bone volume fraction (%) of PCL/PLGA/TCP and PCL/PLGA/duck beak scaffold groups was significantly higher than those of control and PCL/PLGA scaffold groups. The values are the mean ± SD (*n* = 4). ^*∗*^Significantly different (*P* < 0.05) from PCL/PLGA. ^§^Significantly different (*P* < 0.05) from PCL/PLGA/TCP.

Bone volume fraction (%) of PCL/PLGA/TCP and PCL/PLGA/duck beak scaffold groups was significantly higher than those of control and PCL/PLGA scaffold groups (Figures 4 and 5). Although the PCL/PLGA/duck beak scaffold group showed the highest bone volume fraction compared to other groups, no significant difference was observed compared to PCL/PLGA/TCP scaffold group.

### 3.4. Bone Formation Markers and Oxidative Stress Markers

**Figure 6:**
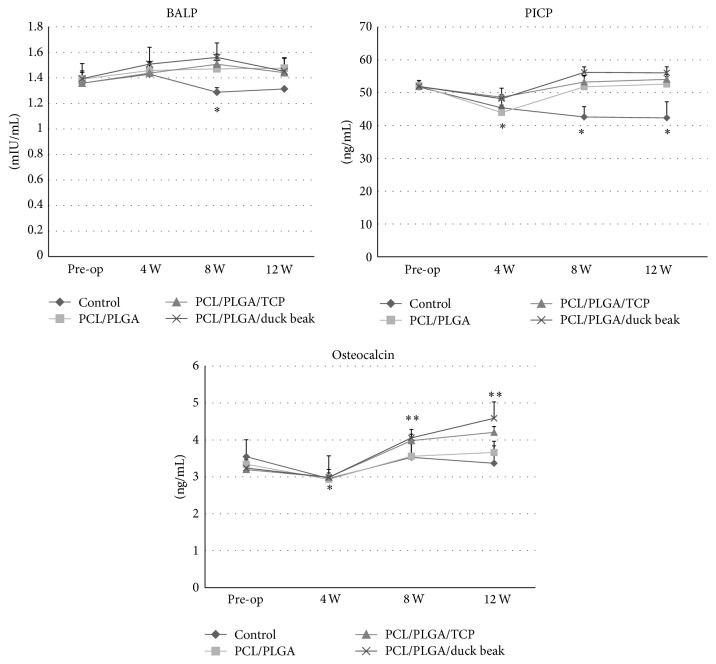
Changes in bone formation markers after implantation. The mean serum BALP, PICP, and OC followed different patterns in control group and scaffold groups. The values are the mean ± SD (*n* = 4). Mean values that are significantly different from pre-op for that group are indicated as *∗*. Mean values that are significantly different from other groups at that time point are indicated as *∗∗* (PCL/PLGA/TCP and PCL/PLGA/duck beak scaffold groups).

**Figure 7:**
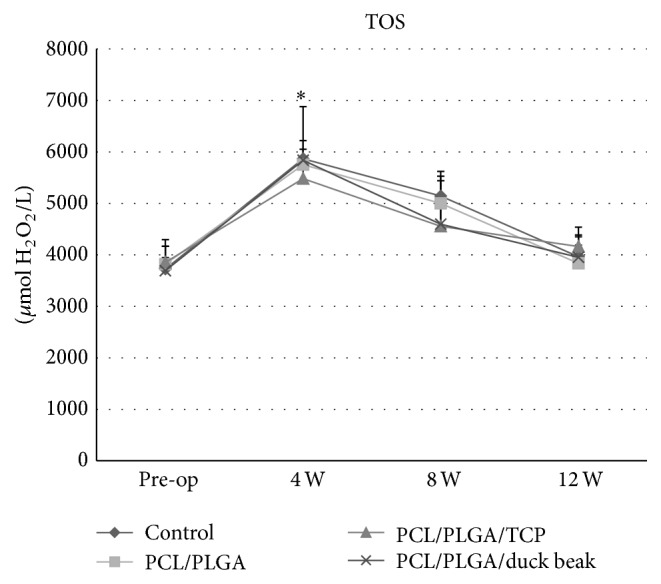
Changes in oxidative stress markers after implantation. The mean serum total oxidant status (TOS) followed the similar pattern in control group and scaffold groups. The values are the mean ± SD (*n* = 4). ^*∗*^Significantly different (*P* < 0.05) from baseline.

The mean serum BALP levels increased above the baseline in all groups, except the control group, and remained unchanged at 12 weeks (Figure 6). The level of serum PICP in control group decreased during the observation period (Figure 6). However, an initial decrease in the peak serum PICP levels was observed in scaffold implantation groups at 4 weeks, followed by a gradual increase over the remaining observation period. The serum OC levels in all groups decreased at 4 weeks but that of scaffold implantation groups increased at 8 and 12 weeks (Figure 6). TOS of all groups significantly increased after surgery (Figure 7). An initial increase in the peak TOS was noted at 4 weeks followed by a gradual decrease over the remaining observation period. No significant difference was observed among the groups.

### 3.5. Histopathological Findings

**Figure 8:**
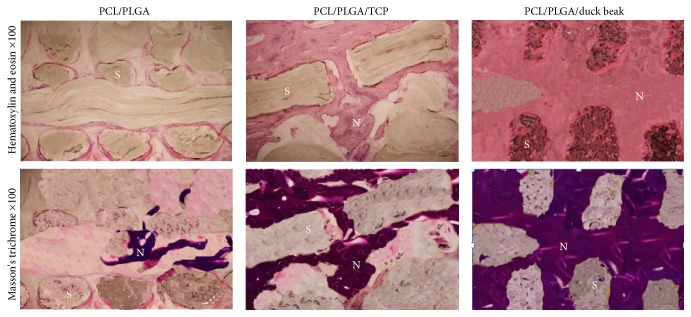
Histological findings of newly formed bone (S: scaffold, N: new bone) in the PCL/PLGA, PCL/PLGA/TCP, and PCL/PLGA/duck beak scaffold groups 12 weeks after surgery. The results revealed advanced progression of new bone formation and mineralization in the PCL/PLGA/duck beak scaffold group compared to the PCL/PLGA and PCL/PLGA/TCP groups. No inflammatory cells noted in any groups.

Histological examination showed that there were numerous new bone matrices and grafted PCL/PLGA/duck beak scaffolds over all areas of the defect sites at 12 weeks after implantation. PCL/PLGA group had less new bone tissue compared to PCL/PLGA/TCP and PCL/PLGA/duck beak scaffold groups at 12 weeks after implantation. At 12 weeks after implantation, bone-remodelling process was complete, and intact bone structures were easily observed in PCL/PLGA/duck beak scaffold group (Figure 8).

## 4. Discussion

In this study, various analytical methods such as X-ray, micro-CT, and histology and biochemical parameters including bone formation and oxidative stress markers confirmed that PCL/PLGA/duck beak scaffolds showed the best bone-healing quality at initial period after implantation without any inflammatory response.

All scaffolds were successfully fabricated from different materials by the MHDS technique for bone formation of critical-sized rabbit segmental diaphyseal defect. A rabbit diaphyseal defect model has been well established in the assessment of biomaterials targeted for bone regeneration applications [14]. Like majority of the studies where the critical-sized defects were used, the present study intends to utilize critical-sized defects to investigate the effects of three types of scaffold. The study aimed to compare scaffolds with varying blended polymer scaffolds and to determine the effects of the biochemical properties on the bone-healing patterns.

The development of specific and sensitive biochemical markers, such as BALP, PICP, and OC, has markedly improved the assessment of bone turnover in various metabolic bone diseases [15]. These biomarkers are useful in monitoring the treatment efficacy in patients with bone fracture and osteoporosis [16, 17]. Additionally, the biochemical, immunohistochemical, histological, and radiological appearances of callus in the experimental animals showed that high levels of OC promoted the healing of bone fractures [18]. Here, OC levels significantly increased in the PCL/PLGA/TCP and PCL/PLGA/duck beak scaffold groups at 12 weeks after implantation compared with those of control group. Hence, elevated OC levels might increase the new bone formation and consequently could accelerate healing of bone fractures, since high levels of OC and BALP are positively correlated with the new bone formation [16].

Various studies linked tissue damage caused by oxygen free radicals to ischemia-reperfusion mechanism [10, 19, 20]. Göktürk et al. evaluated oxidant status in bone specimens during bone healing in rats using malonyldialdehyde (MDA) levels as an indicator of oxidative stress [21]. They observed statistically significant increase in oxidative stress status on days 7 and 14 after experimentally fracturing the right tibia of the study rats, concluding that oxidative stress occurs during the 2nd and 3rd weeks after a fracture. Here, we evaluated the oxidative stress status during bone healing in rabbits by determining the TOS in plasma. Plasma TOS was used to reflect overall oxidative stress. An evaluation of TOS can indirectly reflect changes in organ microcirculation [13]. The present study showed that the mean TOS of the experimental groups was significantly higher than that of the baseline in all group. The mean serum total oxidant status (TOS) followed the similar pattern in control group and scaffold groups. These data suggest that oxidative stress during fracture healing is greatest in the inflammation period. The fact that TOS in the experimental group was significantly different from the control group in the following days indicates that oxidative stress also persists during the repair period, although it is not as great as that during the inflammation period. Inflammatory cells and osteoclasts produce reactive oxygen free radicals [9]. Hence, inflammatory cells and osteoclasts have important roles in generating oxidative stress in the early periods of the bone fracture healing.

Here we used X-ray, micro-CT, and histological methods to analyse bone-healing patterns. X-ray images were used to determine the bone-healing scores. Micro-CT was employed to provide an accurate means to quantify bone and its spatial growth and 3D distribution. Histology images were used to determine the qualitative aspects of bone formation, vascular, cellular, and inflammatory activities. Micro-CT data revealed that PCL/PLGA/duck beak scaffolds showed superior bone formation as compared to the other groups throughout the experiment. Histological analyses revealed increased amount of new bone formation in PCL/PLGA/duck beak scaffolds. The presence of intense inflammatory cells or fibrous encapsulation was not detected.

## 5. Conclusions

In the present study, we developed a novel PCL/PLGA/duck beak scaffold using MHDS technology. X-ray, micro-CT, biochemistry, and histological analyses revealed that the PCL/PLGA/duck beak scaffold promotes new bone formation in rabbit radius by promoting the repair process, suggesting that PCL/PLGA/duck beak scaffold may be a good option for the treatment of fracture.
